# Research on Wind Field Correction Method Integrating Position Information and Proxy Divergence

**DOI:** 10.3390/biomimetics10100651

**Published:** 2025-10-01

**Authors:** Jianhong Gan, Mengjia Zhang, Cen Gao, Peiyang Wei, Zhibin Li, Chunjiang Wu

**Affiliations:** 1 College of Software Engineering, Chengdu University of Information Technology, Chengdu 610225, China; gjh@cuit.edu.cn (J.G.); 3230707020@stu.cuit.edu.cn (M.Z.); weipy@cuit.edu.cn (P.W.); lizhibin@cuit.edu.cn (Z.L.); westbuddha@cuit.edu.cn (C.W.); 2 Sichuan Key Laboratory of Software Automatic Generation and Intelligent Service, Chengdu University of Information Technology, Chengdu 610225, China; 3 Key Laboratory of Meteorological Software China Meteorological Administration, Chengdu 610225, China; 4 Dazhou Key Laboratory of Government Data Security, Sichuan University of Arts and Science, Dazhou 635000, China; 5 Observation Centre, China Meteorological Administration, Beijing 100081, China

**Keywords:** wind field correction, position information, attention mechanism, physical consistency, pointnet, hyperparameter optimization

## Abstract

The accuracy of numerical model outputs strongly depends on the quality of the initial wind field, yet ground observation data are typically sparse and provide incomplete spatial coverage. More importantly, many current mainstream correction models rely on reanalysis grid datasets like ERA5 as the true value, which relies on interpolation calculation, which directly affects the accuracy of the correction results. To address these issues, we propose a new deep learning model, PPWNet. The model directly uses sparse and discretely distributed observation data as the true value, which integrates observation point positions and a physical consistency term to achieve a high-precision corrected wind field. The model design is inspired by biological intelligence. First, observation point positions are encoded as input and observation values are included in the loss function. Second, a parallel dual-branch DenseInception network is employed to extract multi-scale grid features, simulating the hierarchical processing of the biological visual system. Meanwhile, PPWNet references the PointNet architecture and introduces an attention mechanism to efficiently extract features from sparse and irregular observation positions. This mechanism reflects the selective focus of cognitive functions. Furthermore, this paper incorporates physical knowledge into the model optimization process by adding a learned physical consistency term to the loss function, ensuring that the corrected results not only approximate the observations but also adhere to physical laws. Finally, hyperparameters are automatically tuned using the Bayesian TPE algorithm. Experiments demonstrate that PPWNet outperforms both traditional and existing deep learning methods. It reduces the MAE by 38.65% and the RMSE by 28.93%. The corrected wind field shows better agreement with observations in both wind speed and direction, confirming the effectiveness of incorporating position information and a physics-informed approach into deep learning-based wind field correction.

## 1. Introduction

With the transformation of the global energy structure, wind energy has garnered significant attention as a clean and renewable energy source. Among its applications, wind field data plays a crucial role in meteorological forecasting, wind energy utilization, environmental monitoring, aviation safety, and other fields, making accurate wind field data analysis of utmost importance. However, because of the limitations of observational methods and technologies, traditional wind field data primarily originate from single high-dimensional data sources. Single data sources, which may suffer from issues such as incomplete spatial coverage, insufficient resolution, and limited accuracy, make it challenging to fully exploit these multidimensional, multi-source data, thereby restricting the utilization of wind field data.

Although several numerical wind field products are currently available, the majority are limited to capturing mesoscale weather and simulating the mean state of the wind field. Furthermore, deviations in the initial conditions of the dynamical processes, compounded by inherent model uncertainties, prevent current forecast products from accurately resolving detailed wind field distribution information at the surface and for nowcasting applications [1]. In addition, although ground observation data like radar and sounding data provide high resolution, their sparse distribution and susceptibility to terrain effects significantly constrain their coverage. Therefore, a key challenge in current research is to effectively integrate data from multiple sources to produce high-resolution and high-accuracy wind field outputs. This challenge is similar to that faced by biological organisms in nature. In order to develop a coherent and accurate perception of their environment, biological systems must constantly integrate information from multiple senses (e.g., visual, auditory, tactile) of varying quality and scale. Inspired by this natural efficiency, our research aims to develop a computational framework to mimic adaptive and multiscale information fusion strategies observed in biological systems to address challenges in the fusion of weather data.

Wind field correction techniques are designed to adjust wind speed and direction measurements to eliminate computational errors and improve data quality. Domestically and internationally, researchers have conducted a series of studies to achieve wind field correction and have achieved rich results [2,3]. Traditional approaches are categorized mainly into statistical methods [4,5,6], dynamical methods [7], and conventional machine learning. However, statistical and dynamical methods often struggle to handle structurally complex data and the nonlinear relationships between meteorological variables. In contrast, traditional machine learning methods such as LASSO regression [8], random forests [9], and LightGBM [10] have achieved better results due to their ability to handle these nonlinearities. For example, Guo et al. [11] developed a Stacking ensemble learning model to improve wind speed predictions from the WRF model [12] for Zhengzhou City. Although their method effectively reduced prediction errors, ensemble learning models are often complex and computationally intensive, making them less practical for resource-limited applications. Moreover, these traditional methods rely heavily on manual feature engineering and prior knowledge. Their performance is limited when dealing with missing, imbalanced, or high-dimensional data, making it difficult to meet the demand for high-precision wind field correction.

In recent years, the rapid development of deep learning has provided a wider range of techniques for correcting meteorological data. Deep learning models can automatically learn complex patterns from data, adaptively transforming it into deep and abstract features through non-linear mappings [13,14]. Methods such as convolutional neural networks (CNNs) [15], their variants like U-Net [16,17], Transformers [18], and other methods have been widely applied in data correction, particularly for meteorological data correction. For instance, Fu et al. [19] developed WindNet, a deep learning model that significantly improved the accuracy of sea surface wind forecasts from the Global Forecast System (GFS). Bouget et al. [20] trained a deep learning model to fuse rainfall radar images with wind speed data generated by weather forecast models for short-term rainfall forecasting, achieving breakthrough improvements in the forecast of rainfall intensity and precipitation areas for severe convective weather. Similarly, T. Kesavavarthini et al. [21] proposed a method using two deep learning algorithms—one-dimensional convolutional neural networks (CNN1Ds) and long short-term memory encoder–decoder (LSTM-ED) neural networks—to correct biases in rainfall data during the southwest monsoon season in India. The results showed that the LSTM-ED algorithm had the smallest computational error output and the best performance. To correct errors in meteorological satellite remote sensing data, Cao et al. [22] developed a deep learning model that is specific to Atmospheric Motion Vectors (AMVs). Their experimental results showed that the corrected observational errors were significantly reduced, with the root mean square error (RMSE) decreasing by nearly 40% compared to the ERA5 true values.

However, these approaches have limitations. ERA5 is a reanalysis dataset generated through complex data assimilation, not actual observations. Consequently, corrected values inevitably approximate a calculated value, leading to errors and limiting the method’s authenticity in practical applications. Similarly, Zhang et al. [23] developed the MT-DETrajGRU model to correct wind components, but its reliance on ERA5, which has an inherent 3-6 hour latency, can compromise real-time performance. Essentially, using reanalysis data like ERA5 for training amounts to fitting one model to another. Furthermore, these purely data-driven approaches often overlook the underlying physics of climate models. As a result, their corrections may lack physical consistency and fail to accurately represent real-world climate conditions. Recently, many studies have begun to integrate physical principles into deep learning models. For example, Luo et al. [24] proposed a physically guided deep learning framework, PhyDL-NWP, which can predict meteorological variables from arbitrary spatiotemporal coordinates. It calculates physical terms through automatic differentiation and uses physically informed loss to ensure consistency between predictions and control dynamics. By integrating physical equations and potential force parameters into data-driven models, PhyDL-NWP improves weather system forecast performance and physical consistency. Wi et al. [25] proposed a physically constrained deep learning model, MC-LSTM-PET, which enforces a constraint through architectural design to limit evapotranspiration loss to no more than potential evapotranspiration (PET), significantly enhancing physical plausibility. Its runoff response aligns with traditional process models (−6% to −9% runoff reduction).

To address the issues mentioned above, this paper proposes PPWNet, a deep learning model for wind field correction that integrates positional information and a divergence-informed loss term. The model employs a dual-branch DenseInception architecture to extract multiscale spatial features from grid data at different resolutions, which is similar to the biological visual hierarchical processing mechanism, and can perceive macroscopic holistic information and microscopic local details simultaneously. For sparse and discretely distributed sounding point data, the model draws inspiration from the PointNet architecture and incorporates an attention mechanism, enabling it to adaptively assess the importance of each observation point and assign higher weights to critical points. This addresses the challenge of effectively utilizing discrete sparse data that traditional methods struggle with. PPWNet also incorporates physical knowledge into the model training process by adding divergence to the loss function, ensuring that the correction results approximate the observed true values and comply with objective physical laws. Additionally, neighborhood loss is incorporated into the loss function to enhance the spatial continuity and smoothness of the predicted wind field. This hybrid optimization strategy, which combines data-driven approaches, a physics-informed term, and spatial smoothness considerations, significantly improves the overall quality and reliability of the prediction results.

The main contributions and innovations of this paper are summarized as follows:

(1) A correction model (PPWNet) is proposed based on the geographical location information of observation positions and meteorological grid data. This model can effectively integrate grid data of different resolutions and sparse, irregular sounding point observation data to correct wind field data. Crucially, it uses real observational data as the truth values for training.
(2) We introduce a physics-informed term as an optimization strategy for the model. A physics-informed loss term, designed to approximate wind field divergence, is added to the loss function of the model, ensuring that the model improves data fitting accuracy while adhering to the basic principles of fluid dynamics in its prediction results. This enhances the stability and accuracy of the model.
(3) We design a feature extraction and interactive fusion framework for heterogeneous data. For grid data, a dual-branch (DenseInception) extractor combining DenseNet and Inception was constructed to capture multiscale spatial features and enhance feature reuse. For irregularly sounding point data, the PointNet approach was adopted, and an attention mechanism was introduced to extract features and weigh key information for unordered points effectively. Finally, cross-modal information was deeply fused through a feature interaction module.

## 2. Related Work

### 2.1. Multi-Scale Feature Extraction Network

The Inception [26,27] method was proposed by Google. Its core idea is to use multiple convolutional kernels of different sizes (such as 1×1,3×3,5×5) and pooling operations in parallel at the same layer. The outputs from these operations are then concatenated along the channel dimension. This design allows the network to capture features at multiscales from the same input, enabling subsequent layers to select the optimal feature combination. The Densely Connected Network (DenseNet) [28,29] aims to solve the vanishing gradient problem in deep networks and enhance feature reuse. Its key innovation is that each layer receives the feature maps from all preceding layers as its input and passes its own feature maps to all subsequent layers. This “feature reuse” mechanism significantly promotes information flow within the network, achieving superior performance with fewer parameters and fewer computational resources.

The DenseInception module designed in this paper is an organic combination of these two concepts, by embedding Inception’s multi-scale feature extraction capability into DenseNet’s dense connectivity. This design mimics the hierarchical, multi-scale processing strategy of the biological visual system, similar to how the visual cortex uses different clusters of neurons to respond to stimuli of varying scales, from simple edges to complex objects, to form a comprehensive perception, enabling the module to extract features ranging from fine to coarse granularity from grid data; simultaneously, the dense connections ensure that this multi-scale information is effectively retained and not lost during transmission through the deeper layers of the network.

### 2.2. Attention Mechanism

The attention mechanism [30,31] is a neural network technique that mimics human cognitive attention. When processing data like sequences or sets, it allows a model to focus on the most important and relevant parts of the input. The basic principle is to calculate an “importance weight” for each element of the input through an independent network module, and then perform weighted aggregation of the element features based on these weights. In the classic Transformer model [32], the calculation of attention weights is represented in Equation (1):

(1) $$\mathrm{Attention}\left(Q,K,V\right)=\mathrm{softmax}\left(\frac{QK^{⊤}}{\sqrt{d_{k}}}\right)V$$

where *Q*, *K*, and *V* represent the query, key, and value matrices, respectively. In this work, the attention mechanism plays a critical role. It is introduced into the point feature extraction module to enable the model to adaptively evaluate the contribution of each sounding point and assign higher weights to key information.

### 2.3. Physics-Informed Deep Learning

Although deep learning models have powerful data fitting capabilities, their inference results may sometimes violate fundamental physical laws. To address this issue, Mohan et al. [33] proposed a physics-informed neural network (Physics-Informed Neural Network, PINN), whose core idea is to integrate known physical laws, often described by Partial Differential Equations (PDEs), as a soft constraint within the network’s optimization process. The most common implementation adds a physical residual trem to the loss function. The total loss function L is typically made up of a data-driven loss $L_{\mathrm{data}}$ and a physical consistency loss $L_{\mathrm{phys}}$, weighted and calculated as shown in Equation (2):

(2) $$L=L_{\mathrm{data}}+\lambda L_{\mathrm{phys}}$$

where $L_{\mathrm{phys}}$ is used to measure the degree, the model output satisfies the physical equations. By minimizing this residual term, the model is guided to adhere to physical principles while simultaneously fitting the training data.

This idea has been widely applied to enhance the physical realism of data-driven models. For example, He et al. [34] proposed a physical knowledge distillation-based spatio-temporal Transformer network (PKD-STTN) for weather forecasting, which models weather changes based on the potential energy in the atmosphere to reveal the physical mechanisms of atmospheric motion. This paper applies the concept of physics-informed modeling to wind field correction by adding a wind field divergence term to the loss function to guide the model to learn solutions that are not only accurate in terms of data but are also more reasonable in terms of physics.

## 3. Problem Description

The core issue of this paper is how to effectively integrate multi-source wind field data from different sources, resolutions, and structures (e.g., regular grids and irregular discrete points). The goal is to obtain corrected wind field data that is both closer to true observational values and consistent with physical laws. Data from a single source, whether it is a numerical model forecast or a surface observation, has inherent limitations.

The objective of this paper is to learn a function *f* that can receive low-resolution data (ERA5), high-resolution data (high-resolution live data for 1 km (HRLD_1km)), and the spatial coordinates of discrete sounding points P as inputs. It then outputs the u and v components for any given point within the study region. To ensure the model’s output is physically plausible, the wind field divergence is introduced as a physical consistency term. In atmospheric dynamics, in the divergence of the wind field, the divergence is an important physical property. Ideally, the divergence of an incompressible fluid should approach 0. Therefore, a penalty term for the divergence is added to the model’s optimization objective. This guides the model to learn a wind field that is not only numerically accurate but also aligns more reasonably with physical laws.

Ultimately, the model is optimized by a hybrid loss function that combines a data-driven supervised error with a physics-driven consistency penalty.

## 4. Mothods

Given three types of meteorological data—high-resolution gridded data (HRLD_1km), low-resolution gridded data (ERA5), and discrete sounding point data—the task is to compute the u and v wind components of the wind speed at any specified point within the study area. The low-resolution grid data $G_{1}\in R^{H_{1}\times W_{1}\times C}$, where $H_{1}$ and $W_{1}$ are the grid’s height and width, respectively, and C is the feature dimension. The features include longitude, latitude, altitude, pressure, and the u and v wind components. The high-resolution grid data $G_{2}\in R^{H_{2}\times W_{2}\times C}$, with the same features as $G_{1}$, where $H_{2}$ > $H_{1}$ and $W_{2}$ > $W_{1}$. The sounding point data $P\in R^{N\times C}$, where N is the number of sounding points (variable quantity).

The model needs to learn a mapping from the input data to the wind speed at the sounding points: $\hat{y}=f\left(G_{1},G_{2},P_{\mathrm{features}}\right)$, where $\hat{y}=\left(\hat{u},\hat{v}\right)\in R^{N\times 2}$ represents the corrected wind components, and $P_{feature}$ is the feature of the sounding points (excluding the u and v components of wind components). The objective is to minimize the deviation between the model’s computed values and the true observational data values $y=\left(u,v\right)\in R^{N\times 2}$.

### 4.1. Overall Structure of the Model

The PPWNet model proposed in this paper is designed to fuse and correct multi-source spatial data, specifically combining gridded data with discrete point data. By integrating multi-scale feature extraction, attention mechanisms, and physics-informed loss, the model accurately computes wind components for any given location within the study area. The model’s core process involves several steps.

Firstly, it captures multiscale features from grid data of both high and low resolution. Next, it extracts key features from the unordered, discrete sounding data and applies importance weighting. The model also encodes and applies attention to the neighborhood information of target points. Finally, it fuses all these features, grid, point, positional, and neighborhood features, to generate the prediction results conforming to the physical rules.

**Figure 1:**
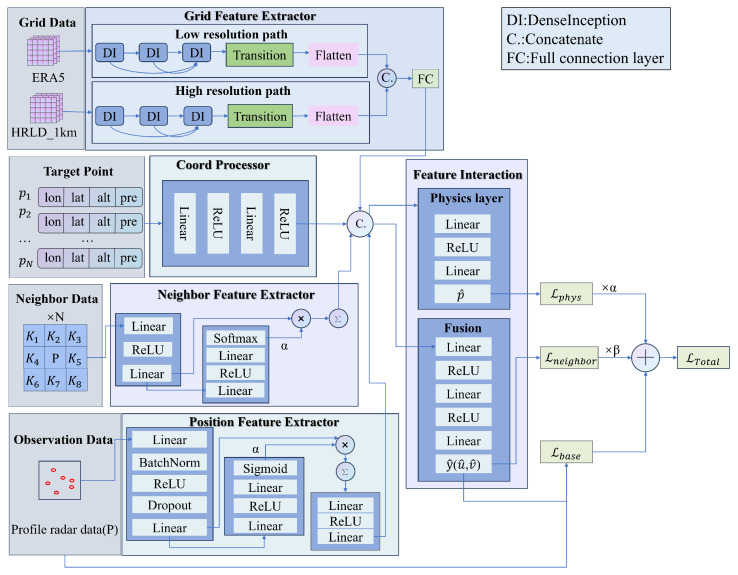
Overall architecture of the PPWNet model.

As shown in Figure 1, the model consists of five core modules: a Grid Feature Extraction Module, a Position Feature Extraction Module, a Coordinate Encoding and Neighborhood Feature Extraction Module, abnd a Feature Interaction and Physical Constraints Module.

### 4.2. Grid Feature Extraction

**Figure 2:**
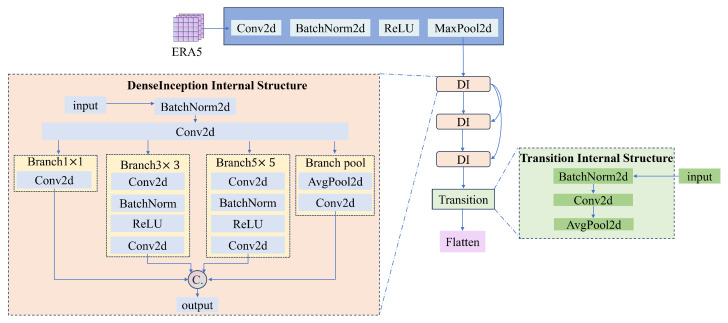
Specific structure of low-resolution branching.

For the feature extraction of grid data with different resolutions, like the low-resolution ERA5 and high-resolution HRLD_1km used in this paper, traditional CNNs struggle to balance the capture of local details in high-resolution data and the control of global trends in low-resolution data. To address this issue, this paper proposes a dual-branch parallel feature extraction architecture. It comprises low-resolution and high-resolution branches, designed to capture spatial features at macro and micro scales, respectively. The specific structure of the branches is shown in Figure 2 (taking the low-resolution path as an example). Each path adopts a DenseInception module, which organically combines the dense connections of DenseNet and the multi-scale feature extraction capabilities of Inception. The input of each branch first passes through a 7 × 7 large kernel convolution layer (stride of 2) to quickly reduce spatial resolution and obtain preliminary feature maps. These feature maps then enter the core network, which is composed of multiple stacked DenseInception blocks. The Inception architecture captures multi-scale spatial features through parallel convolutional branches with different receptive fields. Specifically, it sets up four parallel branches to process the input feature map: a 1 × 1 convolutional branch, a 3 × 3 convolutional branch, a 5 × 5 convolutional branch, and an average pooling branch. To reduce computational costs, the 3 × 3 and 5 × 5 convolutions are preceded by a 1 × 1 convolution for channel dimension reduction. The outputs of all branches are concatenated along the channel dimension, forming a multi-scale feature representation with multiple receptive fields. If the input to a block is $x_{l}$, its output $x_{l+1}$ can be expressed in Equation (3):

(3) $$x_{l+1}=\left[{\mathrm{Conv}}_{1\times 1}\left(x_{l}\right),{\mathrm{Conv}}_{3\times 3}\left(x_{l}\right),{\mathrm{Conv}}_{5\times 5}\left(x_{l}\right),\mathrm{Pool}\left({\mathrm{Conv}}_{1\times 1}\left(x_{l}\right)\right)\right]$$

where [,,,] represents the splicing operation.

To enhance information flow and promote feature reuse, the DenseNet module is introduced. Specifically, multi-scale feature representations are not directly used as inputs to the next layer but are concatenated with the original inputs of the current block. This dense “feature reuse” mechanism ensures information flow from shallow to deep layers, significantly alleviating the vanishing gradient problem and enabling the network to learn richer feature combinations with fewer parameters. This means that the output of each DenseInception block is not only passed to the next block, but also concatenated with the original input, together serving as the input for the next block.

The final output $H_{l}$ of the *l*-th block is shown in Equation (4):

(4) $$H_{l}=T_{l}\left(\left[H_{0},H_{1},\ldots ,H_{l-1}\right]\right)$$

where $\left[H_{0},H_{1},\ldots ,H_{l-1}\right]$ represents the splicing of the output feature maps from layer 0 to layer l−1, and $T_{l}$ is the transformation of the DenseInception block in layer *l*. After several densely connected DenseInception blocks, a transition layer is also included. This layer uses a 1 × 1 convolution to compress the number of channels. It also uses average pooling (AvgPool2d) with a step size of 2 to reduce the spatial resolution of the feature maps. These steps control the model’s complexity and expand the receptive field of subsequent layers.

Eventually, the feature maps extracted from both low-resolution and high-resolution paths are flattened and concatenated into a unified feature vector. This vector is then passed through a fully connected fusion layer to form a final grid feature vector, $F_{grid}$, which simultaneously contains both global trends and local details.

### 4.3. Position Feature Extraction

To effectively utilize observation data from an inconsistent number of irregularly distributed points, this module is inspired by PointNet and is designed to directly process the unordered set of sounding data points, $P=\{p_{1},p_{2},\ldots ,p_{N}\}$. Where each $p_{i}$ contains multiple observation features, in the model n_features = 4. The core of this module is an aggregation strategy based on an attention mechanism. First, each point $p_{i}$ is independently transformed by an MLP with shared weights, which maps the point from the original feature space to a higher-dimensional abstract feature space, yielding the feature $h_{i}=MLP\left(p_{i}\right)$. To distinguish the contributions of points at different positions, an attention mechanism is also introduced into the model. For each point’s abstract feature $h_{i}$, its importance weight $\alpha_{i}$ is calculated through an independent attention network.

It is worth noting that the attention mechanism adopted in this module differs fundamentally from the self-attention commonly found in models like Transformers [32]. Self-attention is designed to handle ordered sequence data such as text, with the core objective of capturing dependencies between elements within a sequence. In contrast, the point feature extraction module in this module is designed for inherently unordered and irregular point sets, and the core advantage of the PointNet approach lies in its permutation invariance, meaning that the global features extracted remain the same regardless of the order of the input points.

The Sigmoid function is used in the model to calculate an independent attention weight $\alpha_{i}$ for each point. The formula is shown in Equation (5):

(5) $$\alpha_{i}=\mathrm{Sigmoid}\left({\mathrm{MLP}}_{\mathrm{attn}}\left(h_{i}\right)\right)$$

This weight depends solely on the characteristics $h_{i}$ of the point itself, measuring the “independent contribution” or “intrinsic importance” of the observation point to the global wind field state. The use of the Sigmoid function means that the weight of each point is calculated independently between [0, 1], and the model can consider multiple points to be equally important or equally unimportant.

In traditional attention, the weights are obtained by calculating the dot product of the query and key and then normalizing it using the Softmax function, as in Equation (1). The attention weight of each point depends on its association with all other points in the set, which is a measure of “relative importance.” The Softmax function forces the sum of all weights to be 1, creating a competitive relationship.

Finally, the features of all points are weighted and summed according to their corresponding attention weights to generate a fixed-length global feature vector $F_{point}$ that can represent the state of the entire observation network. $F_{point}$ is calculated as shown in Equation (6):

(6) $$F_{\mathrm{point}}=\sum_{i=1}^{N}\alpha_{i}h_{i}$$

this vector is then subjected to a further nonlinear transformation by an output MLP to enhance its expressive power.

### 4.4. Coordinate Encoding and Neighborhood Feature Extraction

Grid data points have fixed positions within the study area, whereas the sounding data is sparse and irregularly distributed. Furthermore, some points do not even have values at certain moments because of the observation conditions. Therefore, to emphasize the importance of the location and to provide the model with explicit spatial awareness, we designed a coordinate feature encoding module. This module consists of a fully connected network that receives as input the 3D coordinates (longitude, latitude, elevation) of the target prediction points $X_{coord}=\left(lon,lat,alt\right)$. Through a series of nonlinear transformations, it encodes these raw coordinates into a low-dimensional yet information-rich feature vector $F_{coord}$. $F_{coord}$ is calculated as shown in Equation (7):

(7) $$F_{\mathrm{coord}}={\mathrm{MLP}}_{\mathrm{coord}}\left(X_{\mathrm{coord}}\right)$$

this feature vector provides the model with an explicit coordinate space context that helps the model find the location of points in the grid.

To capture the local spatial information, a neighborhood feature extraction module is designed in the model. This module first identifies the K-nearest neighbors for each target point (where K = 8) based on the 3D Euclidean distance that considers longitude, latitude, and altitude. Subsequently, a neighbor_attention network calculates an importance weight for each of these neighbors. Finally, the encoded features of all neighbor points are then aggregated by weighting to form a comprehensive neighborhood feature vector $F_{neighbor}$.

### 4.5. Constructing the Loss Function

#### 4.5.1. Divergence

In the atmospheric dynamics process, wind field divergence measures the degree to which air converges or diverges horizontally. The wind field is represented by the vector V=(u,v), where *u* is the wind speed in the east–west direction (x-axis) and *v* is the wind speed in the north–south direction (y-axis). The horizontal divergence is calculated by the formula shown in Equation (8):

(8) $$\mathrm{Divergence}=\nabla \cdot V=\frac{\partial u}{\partial x}+\frac{\partial v}{\partial y}$$

where $\frac{\partial u}{\partial x}$ denotes the rate of change of wind speed in the east–west direction, while $\frac{\partial v}{\partial y}$ denotes the rate of change of wind speed in the north–south direction. A common and important approximation in atmospheric science is the assumption of non-divergent flow. This assumes that at certain scales, the wind field divergence is approximately zero (∇·V≈0).

#### 4.5.2. Features Fusion

This module is responsible for making the final prediction by fusing features from all sources. First, the feature vectors $F_{grid}$, $F_{point}$, $F_{coord}$, and $F_{neighbor}$ from the three previous modules are received and spliced in the feature dimensions to form a comprehensive composite feature vector $F_{fused}$ that contains multimodal information. $F_{fused}$ is calculated as shown in Equation (9):

(9) $$F_{\mathrm{fused}}=F_{\mathrm{grid}}⊕F_{\mathrm{point}}⊕F_{\mathrm{coord}}⊕F_{\mathrm{neighbor}}$$

where ⊕ represents the splicing operation. This vector captures the combined information from the sum of grid features, point features, and coordinate features. Subsequently, $F_{fused}$ is fed into two parallel, fully connected networks: a prediction header and a physical consistency layer. The prediction header is a standard network that regresses the final targets, which are the *u* and *v* components of the wind speed, $\hat{y}=\left(\hat{u},\hat{v}\right)$.

(10) $$\hat{y}={\mathrm{MLP}}_{\mathrm{predict}}\left(F_{\mathrm{fused}}\right)$$

The physical layer is the core of physics-informed approach. Directly computing the wind field divergence within a neural network using numerical differentiation is computationally expensive and highly sensitive to output noise, which can lead to unstable training. To circumvent this, we designed the physics layer not to calculate divergence directly, but to learn a proxy residual, $\hat{p}$. The role of this proxy residual, $\hat{p}$, is to serve as a learnable measure of the deviation between the model’s output wind field and physical plausibility. The layer takes the fused feature vector $F_{fused}$ as input and outputs a scalar residual, $\hat{p}$. The formula for $\hat{p}$ is shown in Equation (11):

(11) $$\begin{array}{cc} \hat{p} & ={\mathrm{MLP}}_{\mathrm{physics}}\left(F_{\mathrm{fused}}\right) \end{array}$$

The training objective is to make the proxy residual $\hat{p}$ head towards zero. By penalizing its magnitude in the loss function, the model is indirectly guided to generate a wind field that has a smaller divergence and is more consistent with the physical laws of fluid dynamics, without ever needing to compute the derivatives explicitly.

#### 4.5.3. Loss Function

To effectively train the PPWNet model, a hybrid loss function is designed. It not only measures the discrepancy between the predicted and true values but also introduces a physical consistency term and spatial neighborhood consistency as regularization terms. This approach guides the model to generate predictions that are more physically plausible and spatially smooth. The total loss function is defined as shown in Equation (12):

(12) $$L_{\mathrm{total}}=L_{\mathrm{base}}+\alpha \cdot L_{\mathrm{phys}}+\beta \cdot L_{\mathrm{neighbor}}$$

where α and β are hyperparameters used to balance the importance of the three loss components.

The base loss ($L_{base}$) is the model’s main supervisory signal, which penalizes the error between the model inference wind speed component $\hat{y}$ and the true observation *y*. SmoothL1Loss is adopted as the base loss function. Compared to the L2 loss, SmoothL1Loss is less sensitive to outliers. At the same time, it behaves as smoothly as the L2 loss when the computational error is close to zero. This helps prevent exploding gradients and makes the training process more stable.

The physical loss, $L_{phys}$, is intended to encourage the model’s prediction to be physically realistic. As previously mentioned, instead of direct differentiation, we penalize the proxy residual $\hat{p}$ output by the physics layer. This loss term is defined as the squared L2 norm of $\hat{p}$, and its formula is shown in Equation (13):

(13) $$\begin{array}{cc} L_{\mathrm{phys}} & =∥\hat{p}{∥}^{2} \end{array}$$

minimizing $L_{phys}$ pushes $\hat{p}$ towards 0, indirectly guiding the model to generate a wind field, $\hat{y}$, which is more physically plausible.

The neighborhood loss ($L_{neighbor}$) is designed to enhance the spatial continuity and consistency of the results. This loss term reduces the discrepancy between predictions and observations in the local area around a target point. Specifically, for any target point $p_{i}$, the model first identifies its K-nearest neighboring grid points, denoted as the set $\left\{q_{i,1},q_{i,2},\ldots ,q_{i,K}\right\}$. $L_{neighbor}$ is defined as the average error between the predicted value ${\hat{y}}_{i,j}$ and the corresponding true observed value $\;y_{qi,j}$ of the target point. To enhance the stability of model training and reduce sensitivity to outliers, we use SmoothL1Loss for calculation. For a single target point $p_{i}$, its neighborhood loss is the average loss between it and its K-nearest neighboring grid points, calculated as shown in Equation (14):

(14) $$\begin{array}{cc} L_{\mathrm{neighbor}}\left(p_{i}\right) & =\frac{1}{K}\sum_{j=1}^{K}L_{\mathrm{SmoothL}1\mathrm{Loss}}\left({\hat{y}}_{q_{i,j}},y_{q_{i,j}}\right) \end{array}$$

this encourages the model to generate a smooth wind field that does not vary drastically in the local area, reducing artifacts and unrealistic abrupt changes.

### 4.6. Hyperparametric Optimization Algorithm

Hyperparameters have a significant impact on the model training process, and a reasonable combination of hyperparameters allows the model to converge quickly and generalize well. The goal of hyperparameter tuning is to find the optimal hyperparameter combinations of settings. Traditional manual tuning is a laborious and time-consuming process that relies heavily on expertise. This paper uses the tree-structured Parzen estimator (TPE), a Bayesian optimization algorithm proposed by Bergstra et al. [35] in 2011. The essence of the TPE is to model the relationship between the hyperparameters and the performance of the model by a probabilistic model, and to efficiently search for optimal hyperparameters based on it.

During model training, the TPE iteratively suggests new hyperparameter sets to evaluate. In this work, the key hyperparameters tuned include learning_rate, batch_size, alpha (the physics-informed weight), beta (the neighbor weight), and weight_decay. To efficiently find the optimal solution in the complex parameter space, the TPE algorithm is used in this paper to provide a powerful framework based on Bayesian ideas. The TPE first defines a performance threshold $y^{*}$ based on the existing trial results through a predefined quantile threshold γ. All historical test results are categorized into two groups based on their loss values *y* versus $y^{*}$. The formula is shown in Equation (15):

(15) $$p\left(x|y\right)=\left\{\begin{array}{cc} l(x) & \mathrm{if}\;y<y^{*}\\ g(x) & \mathrm{if}\;y\geq y^{*} \end{array}\right.$$

where l(x) is the distribution of hyperparameters for the group with excellent results, and g(x) is the distribution learned from the configurations that lead to poor performance. The TPE algorithm derives its name from using a tree-structured Parzen estimator to construct l(x) and g(x). The Parzen estimator is a kernel density estimation (KDE) technique. In the estimation (KDE) technique, for each set of one-dimensional data points $\left\{x^{\left(1\right)},x^{\left(2\right)},\ldots ,x^{\left(N\right)}\right\}$, its generalized formula for kernel density estimation is shown in Equation (16):

(16) $${\hat{f}}_{h}\left(x\right)=\frac{1}{Nh}\sum_{i=1}^{N}K\left(\frac{x-x^{\left(i\right)}}{h}\right)$$

where *N* is the total number of data points, *K* is the kernel function, *h* is the bandwidth, which controls the smoothness of the estimated curve, and $x^{\left(i\right)}$ is a hyperparameter point in the historical observation. In the TPE, l(x) and g(x) are Gaussian Mixture Models (GMMs) constructed by the above KDE method using the hyperparameter data of the “superior” and “inferior” groups, respectively. The TPE uses Expected Improvement (EI) to select the next set of hyperparameters that are most likely to result in a performance improvement, and EI is mathematically defined as shown in Equation (17):

(17) $$EI_{y^{*}}=\int_{-\infty }^{y^{*}}\left(y^{*}-y\right)p\left(y|x\right)\;dy$$

By substitution and a series of mathematical derivations via Bayes’ theorem $p\left(y|x\right)=\frac{p(x|y)p(y)}{p(x)}$, it can be shown that the objective of maximizing EI is equivalent to maximizing the ratio $\frac{l(x)}{g(x)}$. Therefore, the optimization problem to be solved by the TPE in each iteration is shown in Equation (18):

(18) $$x_{\mathrm{next}}=\arg\max_{x}\frac{l(x)}{g(x)}$$

By solving this issue, the TPE strikes a balance between exploration and exploitation, which efficiently guides the entire hyperparameter search process.

The choice of the TPE algorithm over meta-heuristic methods such as the Jaya algorithm or particle swarm optimization was made after careful consideration. The TPE is a form of Bayesian optimization that constructs a probabilistic surrogate model of the objective function (i.e., the relationship between the model’s validation loss and its hyperparameters). This enables it to make more informed decisions when determining the next hyperparameter combination to try, thereby balancing the exploration of the search space with the exploitation of promising regions. For deep learning models like PPWNet, each evaluation requires a full training cycle, resulting in very high computational costs. In such cases, the TPE typically offers higher sample efficiency, enabling it to find the best solution with fewer evaluations. In contrast, many meta-heuristic algorithms are population-based and may require a large number of evaluations to converge, making them less practical for computationally intensive tuning tasks. Therefore, this paper selects the TPE to efficiently explore optimal solution combinations in complex hyperparameter spaces while minimizing computational burden.

## 5. Experiments

### 5.1. Dataset

**Figure 3:**
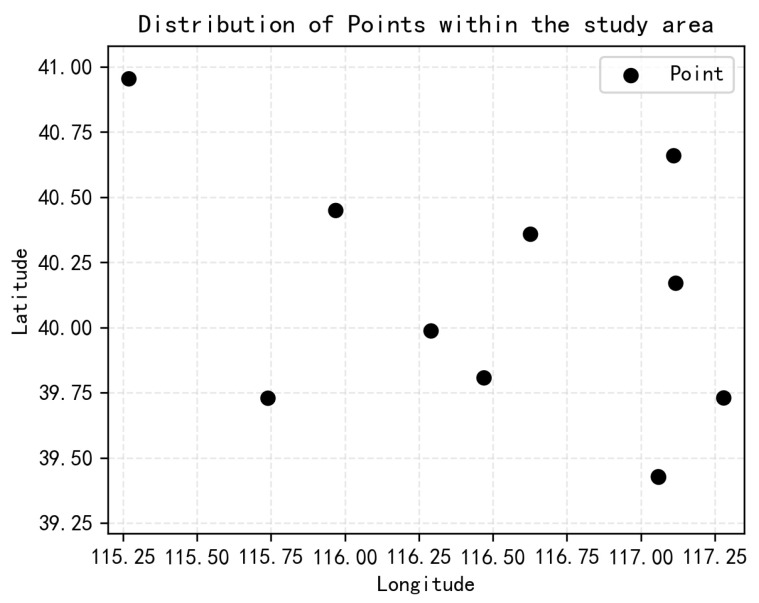
Distribution of probe data locations within the study area.

The data used in this paper are the 1KM high-precision live analysis field data at a regional scale(HRLD_km), the ERA5 data at 0.25° of resolution, the ERA5 dataset, which is the global reanalysis data provided by the European Center for Medium-Range Weather Forecasts (ECMWF), and the wind profile radar sounding data at 11 locations within the region, and the distribution of the sounding data locations within the study area is shown in Figure 3.

The time range of the data is from 00:00 on 1 July 2023 to 23:00 on 31 December 2023, with a time step of 1 h. It is important to note that the dataset is not a continuous time series; there are significant temporal gaps, with numerous hourly time steps missing. Furthermore, for any given time stamp, the data from the 11 observation sites are sparse and incomplete, with a variable subset of stations (typically 6–7) reporting at any one time. Given that our model is designed to correct independent spatio-temporal snapshots and does not rely on temporal sequences, each available time stamp was treated as an independent sample.

To comprehensively evaluate the model’s performance, we employed two distinct data splitting strategies, each serving a specific purpose. The primary method used for model comparison and ablation studies was a random split. The dataset was randomly divided into a training set, a validation set, and a test set in the ratio of 7:2:1. Given that our model is designed to correct independent spatio-temporal snapshots rather than forecasting time-series sequences, this approach effectively assesses the model’s overall generalization and interpolation capabilities across the entire six-month data distribution.

To specifically address the potential for temporal data leakage and evaluate the model’s performance in a more realistic forecasting scenario, we also implemented a chronological holdout (time-blocked) split. For this secondary validation, we designated the final month of our data (December 2023) as the test set, using the preceding data from July to November 2023 for training and validation.

### 5.2. Experimental Setup

#### 5.2.1. Experimental Environment

The hardware configurations used for the experiment are as follows: the central processing unit (CPU) is 16 cores and 32 threads, with a main frequency of 2.60 GHz; the memory is 64 GB, and the storage space is 5 TB. As for the graphics cards, the system is configured with two NVIDIA A5000, each card is equipped with 24 GB of graphics memory, and a total of 48 GB.

#### 5.2.2. Evaluation Metrics

This experiment uses the mean absolute error (MAE), Root Mean Squared Error (RMSE), and coefficient of determination ($R^{2}$) as evaluation metrics. Among them, MAE refers to the average of the absolute value of the difference between the predicted value and the true value, reflecting the average error magnitude of the model prediction. Its calculation formula is shown in Equation (19):

(19) $$\mathrm{MAE}=\frac{1}{n}\sum_{i=1}^{n}\left|y_{i}-{\hat{y}}_{i}\right|$$

RMSE refers to the square root of the mean of the squares of the differences between the predicted and true values, emphasizing the penalty for large errors. It is calculated by the formula shown in Equation (20):

(20) $$\mathrm{RMSE}=\sqrt{\frac{1}{n}\sum_{i=1}^{n}{\left(y_{i}-{\hat{y}}_{i}\right)}^{2}}$$

$R^{2}$ represents the proportion of the variance of the data explained by the model to the total variance, reflecting the model’s ability to fit the data. The formula is shown in Equation (21):

(21) $$R^{2}=1-\frac{\sum_{i=1}^{n}{\left(y_{i}-{\hat{y}}_{i}\right)}^{2}}{\sum_{i=1}^{n}{\left(y_{i}-\bar{y}\right)}^{2}}$$

where n is the number of samples, $y_{i}$ is the actual value, ${\hat{y}}_{i}$ is the predicted value, and $\bar{y}$ is the average of the actual values.

In this experiment, the PyTorch (version 2.1.0) framework is used for model building and training, and the values of hyperparameters are adjusted according to the experiment. The specific hyperparameter values are shown in Table 1.

### 5.3. Analysis of PPWNet Experiment Results

The wind speed component u represents the component in the latitudinal direction (east–west). Positive values represent westerly winds (blowing from west to east) and negative values represent easterly winds (blowing from east to west). The wind speed component v represents the component in the longitudinal direction (north–south). A positive value indicates a southerly wind (blowing from south to north) and a negative value indicates a northerly wind (blowing from north to south). The wind speed is the combined velocity of these two components and is calculated by the formula as shown in Equation (22):

(22) $$\mathrm{Speed}=\sqrt{u^{2}+v^{2}}$$

The wind direction is calculated according to meteorological convention (the direction from which the wind is blowing, measured clockwise from 0° at true north). This can be derived from the standard arctan2(u,v) function, where u and v are the zonal (west-to-east) and meridional (south-to-north) components of the wind, respectively. The formula is shown in Equation (23):

(23) $$\mathrm{Direction}=\left(270-\frac{180}{\pi }\cdot \arctan2\left(v,u\right)\right)\;\left(\mathrm{mod}\;360\right)$$

**Figure 4:**
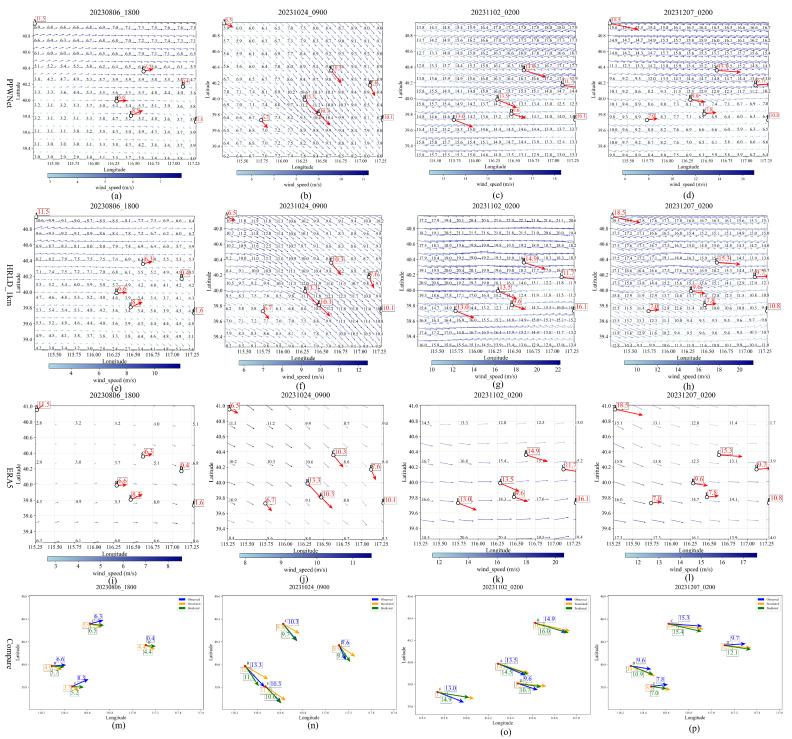
Wind field revisions for the PPWNet model. (**a**–**d**) Corrected wind fields by PPWNet; (**e**–**h**) Original uncorrected HRLD_1km wind fields; (**i**–**l**) ERA5 reanalysis wind fields; (**m**–**p**) Detailed comparison at sounding points A–F.

To visualize the effectiveness of the PPWNet model for wind field correction, the results of four different dates randomly selected from the test results are visualized in the form of vector arrows. As shown in Figure 4, the results of wind field revisions at four moments, on August 6, 2023, at 02:00, are presented in the figure. Figure 4a–d show the 0.01° resolution wind fields corrected by PPWNet, Figure 4e–h display the original, uncorrected HRLD_1km wind fields, and Figure 4i–l show the ERA5 reanalysis wind fields.

In Figure 4, small circles labeled A through F indicate the locations of sounding points. The red numbers represent wind speed, and the direction of the arrows represents the wind direction. For a more detailed comparison, in Figure 4m–p and Table 2, focus on the results at these sounding points and their nearest corresponding grid points. (Note: A comparison with the nearest ERA5 grid point was omitted, as its coarse 0.25° resolution is unsuitable for this fine-scale analysis.)

The blue, orange, and green arrows in Figure 4m–p represent the observed values of wind speed and direction at the sounding point location, HRLD_1km, and the revised values from the PPWNet model, respectively. Figure 4m–p show the location of the sounding point and the wind field on the closest grid point to the point at each of the four time points, chosen to be more centrally located within the study area. A smaller angle between an arrow and the blue reference arrow indicates a more accurate wind direction. The accompanying numbers represent wind speed.

Based on the results shown in Figure 4 and Table 2, the revised wind field using the PPWNet model is numerically and directionally closer to the observed real data in the vast majority of the ranges of the results at the four selected time points. Still, near the boundary of the study area, there are modelled results that are not as close to the real value as the 1km high-precision live analysis field. The reason for this may be that the same location does not always have sounding true value data at different times, as in Figure 4a,c, where point A at the location of Figure 4a does not have a true value in Figure 4c.

### 5.4. Comparative Experiments

In order to verify the effectiveness of the method proposed in this paper, we conduct a comprehensive comparison between the PPWNet model and both traditional approaches as well as several widely used mainstream models. To ensure the rigor and fairness of the comparative experiments, all competing models are trained and evaluated using exactly the same input data as that employed for the PPWNet model. This design guarantees that the differences in performance can be attributed solely to the models themselves rather than variations in the experimental setup.

(1) Kriging Interpolation [36]: Kriging is a spatial interpolation method based on geostatistics, which provides optimal linear unbiased estimation of attribute values at unknown locations by analyzing the spatial correlation of known data points.
(2) LightGBM [37]: LightGBM is a machine learning framework based on Gradient Boosted Decision Trees. It is very suitable for processing tabular data and can mine the complex nonlinear relationships between weather elements and target variables.
(3) U-Net [38]: U-Net is a variant of the classical CNN model, which has a symmetric encoder–decoder structure at its core and fuses shallow features in the encoder with deeper features in the decoder through Skip Connections.
(4) CNN-Transformer [39]: In this architecture, the CNN part is responsible for efficiently extracting the local spatial features in the data, while the Transformer part utilizes its Self-Attention mechanism to capture long-range dependencies and global information in the data.

**Figure 5:**
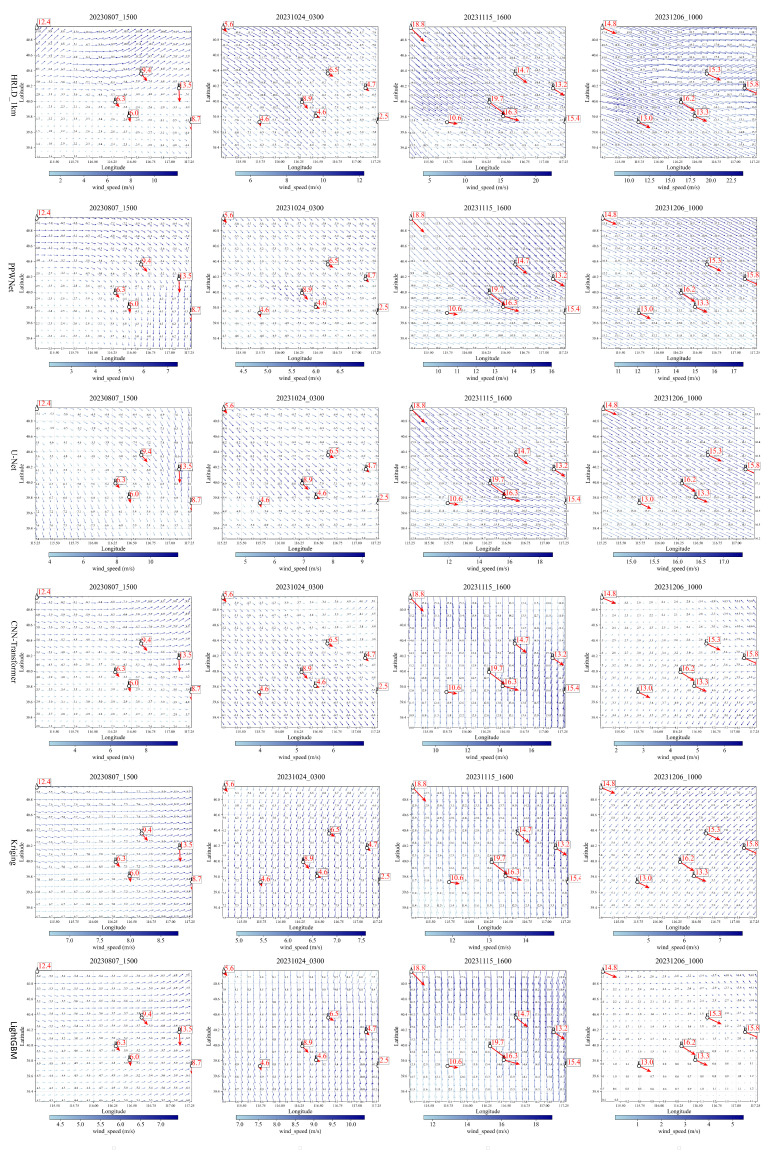
Comparative experimental results. The red number represents the wind speed at that point, and the direction of the arrow represents the wind direction.

The results of the comparison experiments are shown in Figure 5. Among them, the traditional spatial interpolation method, Kriging, performs the worst. This is because it relies solely on sparse sounding data for interpolation and completely ignores the rich background information provided by numerical patterns. The machine learning model LightGBM, while able to incorporate more weather element features, is limited in its performance. It essentially transforms the spatial problem into a tabular regression task, which makes it difficult to capture the spatial structure and local dependencies embedded in the data.

In contrast, the U-Net and CNN-Transformer models show significant advantages over the traditional and machine learning methods, demonstrating the power of deep neural networks in learning complex spatial mappings. U-Net effectively reconstructs high-resolution wind fields through its encoding–decoding structure and skip connections. CNN-Transformer combines the local perception ability of CNNs with the global dependency modelling ability of a Transformer. Compared with each other, the U-Net model is more effective for this research objective of calculating wind field values at any specified point. However, these deep learning models are primarily designed for single gridded data and lack a dedicated mechanism for fusing sparse and irregular data sources like sounding sites.

In contrast, PPWNet demonstrates superior performance due to its specialized architecture. First, it employs a DenseInception-based dual-branch structure to thoroughly extract multiscale features from grid data of varying resolutions. Second, it uniquely processes and fuses sparse sounding data using modules inspired by PointNet, incorporating an attention mechanism. This design allows the model to adaptively focus on the most informative regions and observation positions. As a result, PPWNet can more effectively integrate precise location information into the background wind field, leading to more accurate value predictions at arbitrary points.

To evaluate the computational efficiency of the PPWNet model in practical applications, it was compared with the U-Net and CNN-Transformer models. The evaluation metrics included the number of model parameters, the time required to train each epoch, MAE, RMSE, and $R^{2}$. The results are shown in Table 3.

From the results, PPWNet demonstrates significant advantages in both computational efficiency and prediction accuracy. Compared to U-Net, PPWNet reduces the number of parameters by 74.7% (4.385M vs. 17.367M) and shortens the training time per epoch by 63.3% (6.24s vs. 17.00s), while also outperforming in key prediction metrics: MAE decreased by 16.7% (1.8690 vs. 2.2436), RMSE decreased by 13.9% (3.0526 vs. 3.5447), and R^2^ improved by 1.7 percentage points (0.7324 vs. 0.7200). Compared to CNN-Transformer, PPWNet maintains a 55.5% training speed advantage while improving MAE/RMSE metrics by 38.6%/28.9%, respectively. The experimental results validate the effectiveness of PPWNet’s lightweight design, successfully achieving a synergistic optimization of computational efficiency and prediction accuracy.

### 5.5. Analysis of Dataset Splitting Strategy

To verify whether data leakage exists in the experiments, we analyzed the performance of the model under a strict time series holdout set split and compared it with random split. This analysis aims to validate the model’s temporal generalization capability. The performance metrics of the PPWNet model under both splitting strategies are summarized in Table 4.

As shown in Table 4, the model’s performance is lower on the time-blocked split compared to the random split. This outcome is excepted, as the chronological split represents a more challenging and realistic forecasting task, requiring the model to extrapolate to a future time period with potentially different atmospheric patterns. In contrast, the random split tests the model’s ability to interpolate within a familiar data distribution.

Crucially, even under the stricter evaluation conditions, the PPWNet model maintains robust performance on the chronological test set, achieving an R^2^ of 0.6916. This demonstrates that the model possesses strong generalization capabilities for future, unseen data and is not merely memorizing patterns within the training distribution. This dual analysis not only addresses the concern of data leakage but also confirms that the conclusions drawn from our random split-based experiments are sound and reflect the model’s genuine learning capacity.

### 5.6. Ablation Experiments

To verify the impact of each component on performance, several ablation experiments are designed. The following model variants were evaluated:

- PPWNet-BaseCNN replaces the dual-branch DenseInception module with a standard CNN for grid feature extraction.
- PPWNet-NoAttn removes the attention mechanism from the point feature extraction module.
- PPWNet-NoPhysics removes the physical consistency term by setting the weight of the divergence loss term (α) to zero.
- PPWNet-NoTPE utilizes manually selected hyperparameters based on prior experience, instead of using the TPE optimization algorithm.
- PPWNet is the full proposed model with all components.

**Figure 6:**
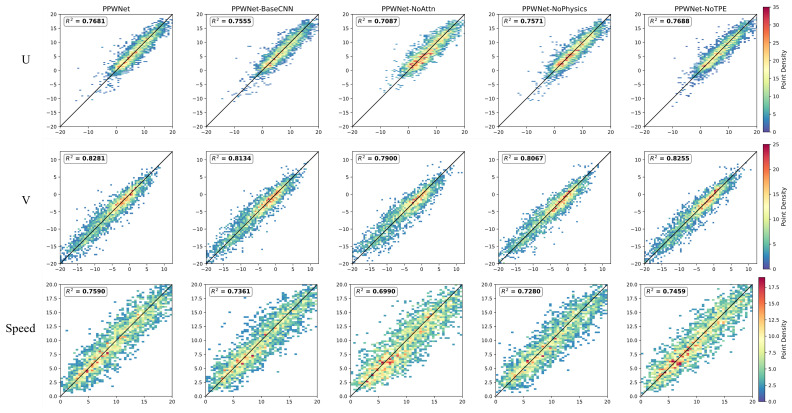
The results of ablation experiments.

The results of the ablation experiment are shown in Figure 6. The results of the evaluation metrics MAE and RMSE are shown in Table 5, where U_MAE, V_MAE, U_RMSE, and V_RMSE represent the MAE and RMSE on the U and V components, respectively.

The experimental results show that every component in the model has some degree of influence on the model’s performance. Specifically, replacing the DenseInception module with the base CNN increases the MAE and RMSE by up to 3.60% and 4.20%, respectively, which suggests that the DenseInception module is better able to obtain more critical information from gridded data with different resolutions through parallel multiscale convolution and dense feature reuse compared to the base CNN; the removal of the attention mechanism has the most significant impact on the model performance, resulting in the highest increase in MAE and RMSE of 16.56% and 12.25%, respectively, which indicates that when processing data with discrete sounding points, the introduction of the attention mechanism allows the model to intelligently assign weights to different points and distinguish which points should be attended to as important information; discarding the physical consistency term increases MAE and RMSE by up to 4.30% and 6.04%, respectively. This indicates that introducing the physical consistency term into the loss function reduces the likelihood of the model generating predictions that go against physical intuition, and thus improves the stability and accuracy of the model.

Furthermore, the comparison between PPWNet and PPWNet-NoTPE demonstrates the practical advantages of automatic hyperparameter optimization. The PPWNet-NoTPE model, tuned manually, still performs well, outperforming models that remove core architectural components. This indicates that the model has a robust underlying structure. However, the PPWNet model optimized using the TPE algorithm achieved superior results across all metrics. As shown in Table 4, compared to PPWNet-NoTPE, PPWNet exhibits reduced values for all evaluation metrics. This shows that while manual tuning can yield a good baseline, automated and systematic search strategies like TPE are more effective in navigating complex high-dimensional hyperparameter spaces, thereby discovering optimal combinations and unlocking the model’s full potential.

It is worth noticing that the $R^{2}$ values presented in the ablation study (Figure 6) may not approach 1; they should be interpreted within the context of the problem domain. Wind field research is an inherently challenging task due to the chaotic and complex nature of atmospheric dynamics. Furthermore, our model tackles the difficult problem of correcting a continuous field using sparse and irregularly distributed ground-truth data. Given these challenges, $R^{2}$ values in the range of 0.70 to 0.83 indicate a strong and meaningful correlation between our model’s predictions and the observed values. The primary goal of the ablation study is to demonstrate the relative contribution of each model component. The consistent degradation in $R^{2}$ and other metrics upon the removal of any component definitively proves the value of our integrated design, even if a perfect forecast remains an elusive goal.

### 5.7. Validation of the Physical Consistency Module

A critical component of our proposed PPWNet architecture is the physical consistency module, which is predicated on the hypothesis that a learned proxy residual, $\hat{p}$, can effectively guide the model toward more physically plausible solutions. To substantiate this core methodological assumption, we conducted a two-part validation study on the held-out test set. The first study establishes the correlation between the proxy and the physical quantity, while the second quantifies the proxy’s efficacy in regularizing the model’s output.

**Figure 7:**
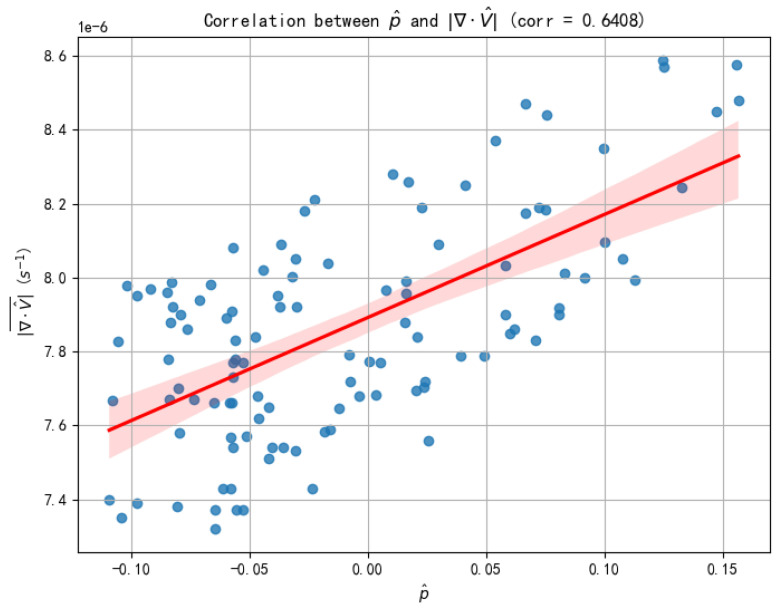
Validation of the physical constraint proxy.

First, we sought to establish a direct empirical link between the learned proxy $\hat{p}$ and true wind field divergence. For each sample in the test set, we computed the divergence of the final predicted wind field, $\nabla \cdot \hat{V}$, using a finite difference method. As illustrated in Figure 7, the analysis of $\hat{p}$ against the absolute divergence $|\nabla \cdot \hat{V}|$ reveals a clear and statistically significant positive correlation (Pearson’s r = 0.6408, *p* < 0.001). This result provides strong quantitative evidence that the physics layer effectively learned to map the model’s internal features to a robust proxy for divergence, thereby validating the foundational premise of our physics-informed loss term.

Second, having confirmed the validity of the proxy, we aimed to quantify its efficacy. To this end, we compared the divergence of predictions from the full PPWNet model against those from the PPWNet-NoPhysics variant, in which the physical consistency loss term was disabled by set the weight α of $L_{phys}$ to zero. The results, summarized in Table 6, show that the inclusion of the physical consistency loss leads to a marked reduction in the mean absolute divergence of the predicted wind field. This demonstrates that our loss term actively and successfully steers the model toward generating outputs that are more consistent with physical principles.

Collectively, these validation studies confirm the dual benefit of our proposed physical consistency module. The learned proxy $\hat{p}$ is a reliable indicator of physical divergence, and its inclusion as a loss term successfully steers the model toward generating wind fields that are not only more accurate but also more physically plausible.

## 6. Conclusions

This paper addresses the challenge of integrating and correcting multi-resolution grid data with sparse, discrete observation positions for wind field correction. It proposes a deep learning model, PPWNet, that integrates positional information with physical principles. The model employs a dual-branch DenseInception module to extract multi-scale grid features, capturing wind field features from both macro (ERA5) and micro (HRLD_1km) scales. This design enhances adaptability across different weather systems; simultaneously, inspired by PointNet and attention mechanisms, a separate module processes sparse, unordered observation positions, enabling wind field prediction at any specified location.

The use of attention mechanisms in model design was proven to be critical for performance improvement, with ablation experiments showing a 16.56% reduction in mean absolute error (MAE) and a 12.25% reduction in root mean square error (RMSE). Furthermore, by incorporating the divergence-informed method—a fundamental law of fluid dynamics—into the loss function, the model not only learns statistical patterns in the data but also adheres to physical principles, enhancing stability and accuracy under varying meteorological conditions. The mean absolute error (MAE) was further reduced by 4.30%, and the RMSE by 6.04%. The training data spans six months and includes diverse meteorological conditions, further ensuring the model’s robustness. Comparative experiments show that PPWNet significantly outperforms traditional methods such as Kriging, machine learning models like LightGBM, and advanced deep learning models like U-Net and CNN-Transformer. These results validate the effectiveness of combining multimodal data with physical principles for wind field correction.

PPWNet demonstrates strong potential for real-world applications, including wind farm management, urban air quality monitoring, and short-term weather forecasting. Its ability to fuse sparse observational data with grid data while adhering to physical laws makes it a strong candidate for deployment in operational meteorological services and renewable energy systems. Future research will focus on adapting the model to different geographical and climatic regions to further enhance its generalization capabilities. Currently, the model is trained on data from a specific geographic region. Therefore, its application to new areas with different terrain or climate characteristics may require validation and fine-tuning to ensure optimal performance. Future research will focus on adapting the model to enhance its generalization across diverse geographical and climatic regions.

## Figures and Tables

**Table 1:** Hyperparameter optimization results.

Hyperparameter	Search Field	Initial Value	Determined Value
Learning rate	($10^{-5}$, $10^{-3}$)	0.0001	0.0002
Alpha	(0.01, 0.5)	0.1	0.0322
Beta	(0.01, 0.5)	0.1	0.0227
Weight decay	($10^{-6}$, $10^{-3}$)	$10^{-5}$	$9.46\times 10^{-6}$
Batch size	[8, 16, 32]	16	32
Epoch	No Search Field	300	300

**Table 2:** Comparison of the location of the sounding point and the wind field of the nearest grid point to the point. S_H, D_H represent the unrevised HRLD_km, respectively, and S_P, D_P represent the values revised by the PPWNet model, respectively.

Time	Point	Speed	Direction	S_H	D_ H	S_P	D_P
20230806_1800	A	11.50	53.00	**11.14**	**71.25**	7.09	74.13
	B	6.60	86.00	4.88	111.98	5.68	**95.07**
	C	6.30	75.00	5.88	103.73	**6.46**	**94.99**
	D	0.40	325.00	**4.39**	113.92	4.44	**94.86**
	E	8.30	66.00	3.33	122.39	**5.22**	**93.86**
	F	1.60	154.01	4.78	136.99	**4.01**	141.51
20231024_0900	A	6.50	113.00	10.75	**99.00**	**6.02**	130.95
	B	13.30	137.00	**12.17**	119.94	11.13	**146.16**
	C	10.30	141.00	8.79	119.92	**9.75**	**141.83**
	D	7.60	155.00	**8.94**	124.78	9.02	**146.45**
	E	10.30	135.00	11.23	124.90	**10.58**	**136.79**
	F	10.10	135.99	**10.40**	**130.78**	8.26	141.30
20231102_0200	A	13.50	113.00	14.78	107.58	**14.47**	**109.51**
	B	14.90	107.00	16.38	102.60	**15.93**	**106.05**
	C	11.70	99.00	16.47	93.94	**16.22**	**100.47**
	D	9.60	108.00	12.96	95.23	**10.67**	**98.31**
	E	16.10	96.00	**16.01**	85.68	13.98	**91.11**
	F	13.00	111.00	17.10	**99.25**	**14.89**	98.88
20231207_0200	A	18.50	102.00	**20.77**	108.83	15.85	**98.67**
	B	9.60	99.00	11.93	112.93	**10.92**	**111.21**
	C	15.30	94.00	16.08	**98.09**	**15.43**	**102.78**
	D	9.70	86.00	12.06	98.56	**12.00**	**94.79**
	E	7.80	83.00	8.83	99.49	**7.01**	**98.25**
	F	10.80	69.00	**8.64**	**72.56**	6.36	75.91

**Table 3:** Performance comparison of different models.

Model	Parameters (M)	Training Time (s/Epoch)	MAE	RMSE	$R^{2}$
PPWNet	4.385	6.24	1.8690	3.0526	0.7324
U-Net	17.367	17.00	2.2436	3.5447	0.7200
CNN-Transformer	14.857	14.02	3.0467	4.2951	0.5863

**Table 4:** Performance Comparison of PPWNet under Random and Time-Blocked Data Splits.

Data Split Method	MAE	RMSE	$R^{2}$
Random Split	1.9022	3.0509	0.7359
Time-Blocked Split	2.3455	3.2640	0.6916

**Table 5:** Ablation study results for different model components, evaluated with MAE and RMSE metrics.

Model	U_MAE	V_MAE	U_RMSE	V_RMSE
PPWNet-BaseCNN	2.0231	1.9946	2.9738	3.0613
PPWNet-NoAttn	2.2654	2.2584	3.2458	3.2469
PPWNet-NoPhysics	2.0252	2.0208	2.9643	3.1154
PPWNet-NoTPE	1.9621	1.9561	2.8959	2.9603
PPWNet	1.9528	1.9375	2.8915	2.9378

**Table 6:** Comparison of Mean Absolute Divergence.

Model	Mean Absolute Divergence (s^−1^)
PPWNet-NoPhysics	8.824765 × 10^−6^
PPWNet	7.226905 × 10^−6^

## Data Availability

Availability of some data is limited. ERA5 data from the European Center for Medium-Range Weather Forecasts (ECMWF) are available with permission from the ECMWF at https://cds.climate.copernicus.eu/datasets/reanalysis-era5-pressure-levels?tab=overview (accessed on 29 November 2024).
